# Identifying Candidate Targets of Immune Responses in Zika Virus Based on Homology to Epitopes in Other Flavivirus Species

**DOI:** 10.1371/currents.outbreaks.9aa2e1fb61b0f632f58a098773008c4b

**Published:** 2016-11-15

**Authors:** Xiaojun Xu, Kerrie Vaughan, Daniela Weiskopf, Alba Grifoni, Michael S. Diamond, Alessandro Sette, Bjoern Peters

**Affiliations:** Division of Vaccine Discovery, La Jolla Institute for Allergy And Immunology, San Diego, California, USA; Division of Vaccine Discovery, La Jolla Institute for Allergy And Immunology, San Diego, California, USA; Division of Vaccine Discovery, La Jolla Institute for Allergy And Immunology, San Diego, California, USA; Department of Medicine, Washington University School of Medicine, Saint Louis, Missouri, USA; Department of Molecular Microbiology, Washington University School of Medicine, Saint Louis, Missouri, USA; Department of Pathology and Immunology, Washington University School of Medicine, Saint Louis, Missouri, USA; The Center for Human Immunology and Immunotherapy Programs, Washington University School of Medicine, Saint Louis, Missouri, USA; Division of Vaccine Discovery, La Jolla Institute for Allergy And Immunology, San Diego, California, USA; Division of Vaccine Discovery, La Jolla Institute for Allergy And Immunology, San Diego, California, USA

## Abstract

Introduction: The current outbreak of Zika virus has resulted in a massive effort to accelerate the development of ZIKV-specific diagnostics and vaccines. These efforts would benefit greatly from the definition of the specific epitope targets of immune responses in ZIKV, but given the relatively recent emergence of ZIKV as a pandemic threat, few such data are available.

Methods: We used a large body of epitope data for other Flaviviruses that was available from the IEDB for a comparative analysis against the ZIKV proteome in order to project targets of immune responses in ZIKV.

Results: We found a significant level of overlap between known antigenic sites from other Flavivirus proteins with residues on the ZIKV polyprotein. The E and NS1 proteins shared functional antibody epitope sites, whereas regions of T cell reactivity were conserved within NS3 and NS5 for ZIKV.

Discussion: Our epitope based analysis provides guidance for which regions of the ZIKV polyprotein are most likely unique targets of ZIKV-specific antibodies, and which targets in ZIKV are most likely to be cross-reactive with other Flavivirus species. These data may therefore provide insights for the development of antibody- and T cell-based ZIKV-specific diagnostics, therapeutics and prophylaxis.

## Introduction

Zika virus (ZIKV) has emerged as a pandemic threat that is associated with severe birth defects^1^^,^^2^^,^^3^^,^^4^^,^^5^. ZIKV is a member of the *Flaviviridae* family, a group of viruses for which many epitopes are known. Because of phylogenetic relatedness, it is likely that some *Flavivirus* epitopes are conserved in ZIKV, possibly contributing to preexisting immunity in areas where ZIKV and other *Flaviviruses*, such as Dengue virus (DENV), West Nile virus (WNV) and Japanese encephalitis virus (JEV) co-circulate. Epitope conservation also raises the possibility that preexisting antibodies to other *Flaviviruses* might enhance ZIKV pathogenesis because of the antibody-dependent enhancement (ADE) phenomenon, whereby antibodies acquired from a previous infection bind, but fail to effectively neutralize the secondary dengue virus serotype, leading to enhanced infection and more severe disease^6^. Finally, epitope sequence identity and viral cross-reactivity pose a challenge to antibody-based diagnostic assays in areas where these viruses co-circulate. Conversely, instances where epitopes map to regions of the *Flavivirus* proteome that are significantly divergent between ZIKV and other *Flaviviruses* also are of interest. If such ZIKV-specific sequences are immunogenic, these epitopes could be of diagnostic value, allowing discrimination between exposure from ZIKV or other co-circulating *Flaviviruses*.

The Immune Epitope Database (IEDB, www.iedb.org)^7^ contains manually curated information from the scientific literature about what specific epitopes are recognized in infectious agents, as well as allergens and auto-antigens. Here, we retrieved the *Flavivirus*-derived data housed in the IEDB related to antibody and T cell epitopes, and compared them to corresponding elements in Zika virus to identify divergent and conserved targets of immune responses in this viral family.

## Methods

**ZIKV sequence database and determination of sequence conservation**

The Entrez package from Biopython was utilized to query the NCBI protein data repository for full-length ZIKV polyprotein sequences, identified as records having taxonomic ID 64320 and length greater than 3,000. These records were then processed to extract associated information (strain/isolate name, accession ID, year, and location). **Supplemental Table 1** lists all ZIKV strains retrieved in this query, representing the set of full-length ZIKV proteomes available as of September 2016. This set includes isolates from the recent outbreak in South America, as well as sequences from previous outbreaks in different geographic locations. Following removal of sequences that occurred more than once, all unique Zika polyprotein sequences were aligned using MAFFT8^8^ . The positional identity and deletion rate were then computed based on the alignment profile to determine sequence conservation.

**Selection of *Flavivirus* Proteome Sequences for comparison to ZIKV**

To analyze sequence conservation among different *Flavivirus* species, the following method was used: For Zika virus, Yellow Fever virus, Japanese encephalitis virus and Tick-borne encephalitis virus a consensus sequence was derived from a multiple sequence alignment of all strains matching the respective taxonomic ID (64320, 11089, 11072, and 11084, respectfully). A BLAST search was then performed using on the consensus sequence to identify a representative strain meeting the following criteria: complete proteome having highest sequence identity to the consensus and full annotation of individual proteins (residue positions). For Dengue virus serotypes 1-4 and West Nile virus, for which many thousands of sequences are available from disparate geographical regions, representative sets of polyprotein sequences were assembled as previously described^9^, in order to prevent regional bias. **Supplemental Table 1** provides the list of all selected strain names, accession IDs, GI numbers, percent match to consensus sequence and country, host and date of isolation, if known. A link to the consensus sequence file is also provided as supplemental material.

**IEDB data curation methodology**

Although the IEDB curation guidelines are detailed elsewhere^10^, we re-iterate some basics here that are relevant to the present analysis. Briefly, the IEDB uses automated document classifiers^11^ to identify all articles indexed in PubMed that describe epitopes. For those scoring above a conservative threshold, the full text articles are retrieved and inspected by a curator who determines if original data specific to epitope recognition is included. One inclusion criterion is that the molecular structure of recognized epitopes was mapped to a region of 50 amino acids or smaller. For antibody responses, this includes linear stretches of amino acids, sets of discontinuous amino acids that form patches in the 3D protein structure, or even single residues, such as those defined by loss of function assays. T cell epitopes always consist of linear amino acid regions and typically span 9-11 amino acids for epitopes recognized by CD8 T cells in the context of MHC class I molecules, and 13-20 amino acids for epitopes recognized by CD4 T cells in the context of MHC class II molecules. As every journal article is curated separately, two epitopes are reported as distinct entities in the IEDB if they have any difference in molecular structures even if they largely overlap. Thus in many cases, the epitopes reported in different studies overlap the same antigenic site.

**IEDB epitope data retrieval**

The IEDB web interface was used to query for all records where the epitope source organism was within the *Flavivirus* genus (NCBI taxonomic ID 11051) for T cell or B cell assays, thus excluding assays only identifying MHC ligands, and included the following fields associated with the records: epitope id, description (sequence), antigen name, position, antigen id (accession) epitope source organism name. For these general analyses, only epitopes identified in human hosts were considered. An exception was made for the ‘functional antibody’ epitope dataset, for which we included epitopes identified in any host species for which the epitope recognizing antibodies were shown to have specific biological functions, namely in vitro neutralization, antibody-dependent cellular cytotoxicity, complement-dependent cytotoxicity and those representing in vivo challenge assays (protection from, survival from challenge, challenge decreased disease and pathogen burden after challenge). This exception was made to take into account that such assays (especially in vivo assays) are essentially exclusively performed in animal model systems.

**Alignment of known epitope data with ZIKV and calculation of RFScore**

Epitope data from *Flaviviruses* extracted from the IEDB were compared to reference ZIKV sequence Rio-U1 [accession: AMQ48981.1]. Each epitope containing *Flavivirus* protein sequence was aligned to the ZIKV polyprotein sequence in order to identify the putative position of the *Flavivirus* epitope in ZIKV. Next, the degree of sequence identity between each epitope and its mapped position on ZIKV was calculated as the percentage of the identical residues in the epitope aligned region. Finally, a positional response frequency (RFscore) was calculated using previously established parameters (http://help.iedb.org/entries/91331263-Immunome-Browser-3-0). Briefly, for a given residue in the protein sequence, data from all epitopes containing that epitope were considered. Assays in which these epitopes were tested were compiled and the number of subjects tested in the assay as well as the number of responding subjects was taken to calculate a frequency of response. To assign a higher weight to sequence regions that were extensively tested and thus have a higher confidence in the calculated frequency of responding donors, the lower bound of the 95% confidence interval associated with that frequency was taken as the RFscore.

**3D Structural Analysis**

Structural analyses were performed using the crystal structures of ZIKV E (PDB 5IZ7)^12^ and NS1 (PDB 5IY3)^13^. Solvent accessibility scores were determined using ASAView: Solvent Accessibility Graphics for proteins (http://www.abren.net/asaview)^14^. 3D renderings were generated using Visual Molecular Dynamics (VMD)^15^. The online interactive 3D visualization of the E and NS1 proteins with SASA, RFscore and Sequence ID mapped onto the structures are implemented using Jsmol, an open source software for interactive 3D viewing of protein structures that uses JavaScript. The 3D rendering of epitopes identified by query of the IEDB was accomplished by mapping *Flavivirus* epitope positions onto the ZIKV proteome.

## Results

**Compilation of Zika virus sequences from NCBI**

We compiled a set of full-length ZIKV sequences by querying the NCBI protein sequence database. **Supplemental Table 1** summarizes the isolate or strain name, accession ID and the year and location of isolation. As of September 2016, 129 ZIKV sequences were retrieved. Of these, 42 are from the 2015-2016 outbreak originating in the Americas. Other available sequences include those from isolates identified in Canada, Europe, Africa, Asia, Southeast Asia, including the original ZIKV isolate MR766 from Uganda in 1947. Twenty-four redundant sequences were removed from this list, resulting in a set 103 unique polyproteins for subsequent analyses.

**ZIKV isolates have high sequence identity**

To assess the degree of variability among different ZIKV isolates, a multiple sequence alignment was performed. Supplemental Figure 1 (see Appendix) displays the degree of conservation for the consensus residue at each position of the three structural proteins capsid (C), prM/membrane (prM/M), envelope (E), and the nonstructural proteins NS1, NS2A, NS2B, NS3, NS4A, NS4B and NS5. Insertions or deletions in the alignment were only found in the E protein. **Table 1** provides the overall sequence identity among the different ZIKV proteins. High conservation was observed across all proteins with an average of 99.2%. Within the ZIKV proteins, slightly greater sequence variability was noted for structural proteins (98.4%) compared to non-structural proteins (99.4%). The greatest sequence divergence was identified within the anchor region of the capsid protein (aa105-122) at 96.8% identity, and the highest conservation in the short non-structural 2K protein (100%).

**Figure:**
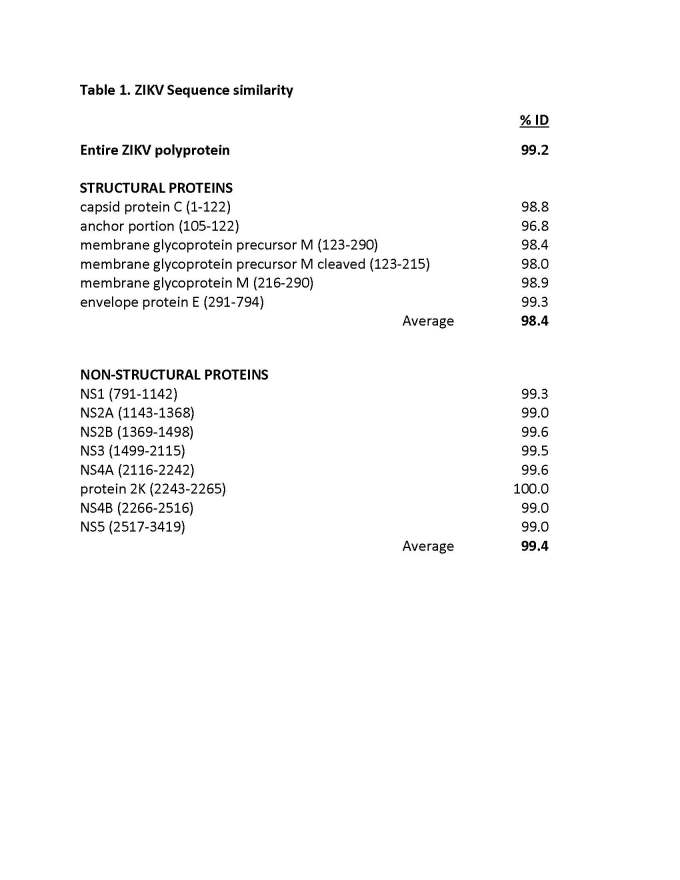
**Table 1.** ZIKV Sequence similarity

As a point of reference, we also computed the sequence variability of isolates from the four DENV serotypes using multiple sequence alignments. Our analysis showed that sequence identity of the polyprotein within DENV serotypes was 98.6%, 98.1%, 98.9% and 99.0% for serotypes 1-4, respectively. As expected, the average degree of polyprotein sequence conservation calculated across the four serotypes was much lower, 83% (range 68.7%-78.1% for DENV1-4 by pairwise alignment). Thus the sequence identity within ZIKV isolates (99.2%) is comparable to the sequence identity of different isolates from a given DENV serotype (98.1 - 99.0%). At the level of polyprotein, the sequence identity between ZIKV and DENV is the highest of all *Flavivirus* species considered herein (55.1%-56.3%). **Table 2** shows the pairwise identities between the reference ZIKV polyprotein and the other selected *Flavivirus* strains.

**Figure:**
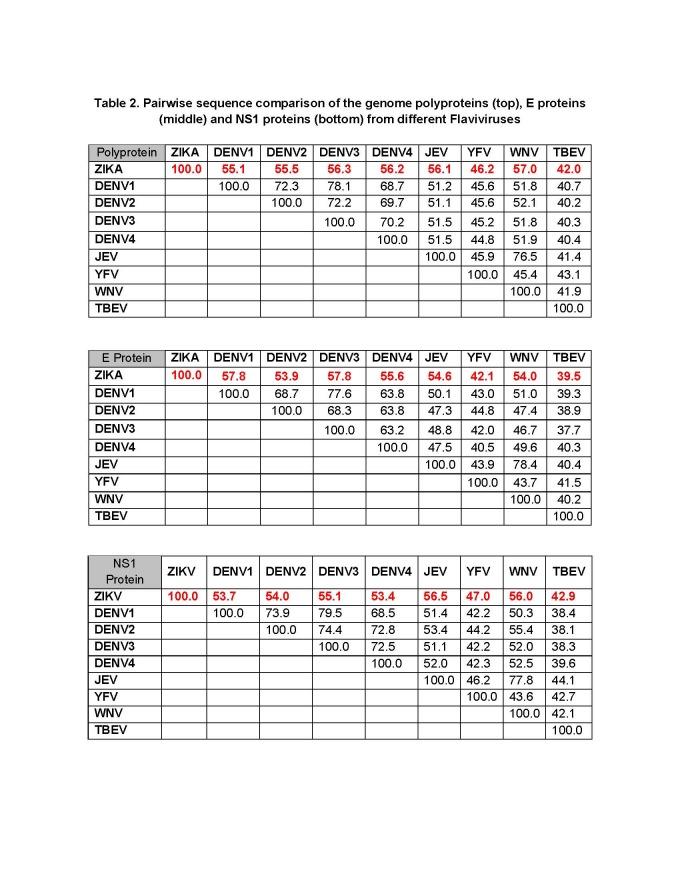
**Table 2.** Pairwise sequence comparison of the genome polyproteins (top), E proteins (middle) and NS1 proteins (bottom) from different *Flaviviruses*

**Retrieval of *Flavivirus* antibody epitopes from the IEDB**

A significant body of literature describes immune targets in *Flaviviruses* other than ZIKV. Of particular relevance for potential serological cross-reactivity are DENV, West Nile virus (WNV), Japanese encephalitis virus (JEV) and Yellow fever virus (YFV), because of their relatively high circulation frequency in the human population. While responses have been observed to all three structural viral proteins C, prM/M, E, and to a lesser extent to the non-structural protein 1 (NS1), the majority of *Flavivirus* antibody responses are thought to be directed against the E protein^16^^,^^17^^,^^18^. Moreover, the E protein is a major target of neutralizing antibodies and has therefore been studied as a source of potential protective epitopes^19^^,^^20^^,^^21^^,^^22^^,^^23^^,^^24^.

To survey the current ‘universe’ of antibody epitopes reported in the literature, we queried the IEDB. The resulting 1,037 *Flavivirus* antibody epitopes defined in more than 3,900 assays, included 502 epitopes defined in humans. **Supplemental Table 2** contains an enhanced export of the entire subset of human antibody epitope data, including positive and negative structures, and provides extensive annotation of these data, including epitope structure and source identification (e.g. linear or discontinuous, protein and strain name), host information (e.g. natural exposure or immunization event) and assay details (e.g. assay type, assay antigen and response frequency). Of the human antibody epitopes that have been defined for *Flaviviruses*, the majority comes from mosquito-borne viruses DENV, WNV, YFV and JEV, with the bulk of data describing DENV epitopes (90%). Antibody epitope data are also available for tick-borne TBEV, which comprise 5% of the overall data. At the level of antigen, as expected, the E protein predominates, comprising >80% of all antibody epitopes. NS1 (6%) accounts for the second largest subset of antibody epitopes, followed more distantly by prM/M (3%), capsid (3%) and NS5 (1.8%). Little or no antibody-specific data are available for NS2A, NS2B, NS3, NS4A and NS4B. Therefore, subsequent analysis of antibody reactivity provided herein emphasizes E and NS1 data defined for humans in these five *Flavivirus* species.

**Homology of the E and NS1 proteins across different *Flavivirus* species**

To investigate the potential for antibody cross-reactivity at the antigen level, we assessed the percent sequence identity of the E protein and the NS1 protein from ZIKV compared to reference strain sequences of other *Flaviviruses* species ****(**Table 2**). For the E protein, we found that TBEV and YFV had the lowest identity (39.5% and 42.1%, respectively) to ZIKV, while WNV, JEV and DENV1-4 had higher sequence identity, ranging from 54.0 to 57.8%. For NS1 protein sequences, we found a similar range of percent identities. Interestingly, the highest NS1 sequence identities were observed for JEV and WNV (56%-56.5%), whereas for the E proteins showed that higher identity with DENV1 and DENV3 (each 57.8%).

To put these data into context, we asked how these sequence identities compared to those between different DENV serotypes, for which there is documented antibody cross-reactivity^25^^,^^26^. Indeed, E proteins from different DENV serotypes had sequence identities at or above 63% (**Table 2**), which is only slightly above the identify levels to between ZIKV and other *Flavivirus* species. Judging based on sequence identity alone it is therefore conceivable that there will be cross-reactive antibody responses between different *Flavivirus* species and ZIKV, as has also been described in the recent literature^27^^,^^28^^,^^29^^,^^30^.

**Alignment of antibody epitope data from other *Flaviviruses* to ZIKV sequences**

To assess which specific antibody targets in the proteins of *Flaviviruses* are conserved in ZIKV and therefore potentially cross-reactive, we generated a plot of epitope sequence identity along the ZIKV reference proteome, overlaid with the response frequency score (RFscore) for each epitope residue (**Fig. 1A, Fig. 1B, Fig. 1C**). The RFscore provides a measure of the relative projected immunogenicity of a given residue by quantifying its reactivity in terms of the number of subjects responding / number subjects tested for all epitopes containing the residue (see Methods).

**Figure 1a:**
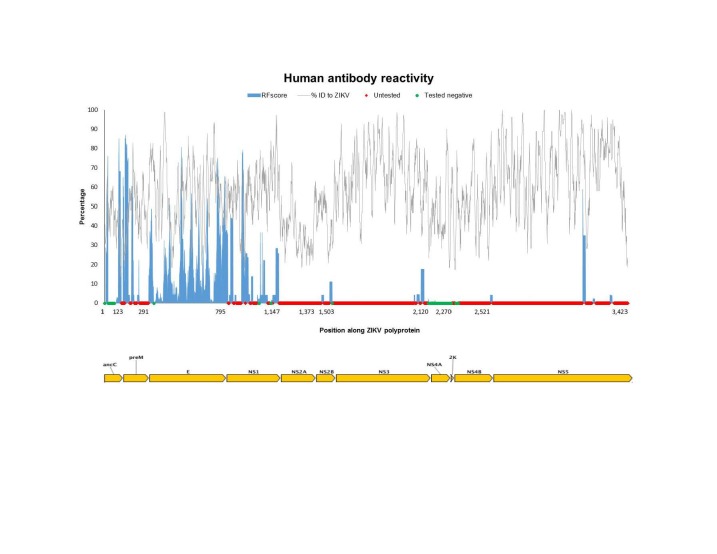
**Fig. 1A:** The Flavivirus sequence identity and human antibody response frequency visualized along the entire ZIKV polyprotein. The sequence identity data (grey) shown represent a running average (window of 9 aa) of sequences for reference Flaviviruses to ZIKV, while the response frequency scores (blue) represent individual residues. Red and green dots along the x-axis represent untested and negative regions, respectively. Residues intervals along the x-axis of the polyprotein plot represent start sites of individual protein products in the polyprotein depicted also at the bottom of panel A.

**Figure 1b:**
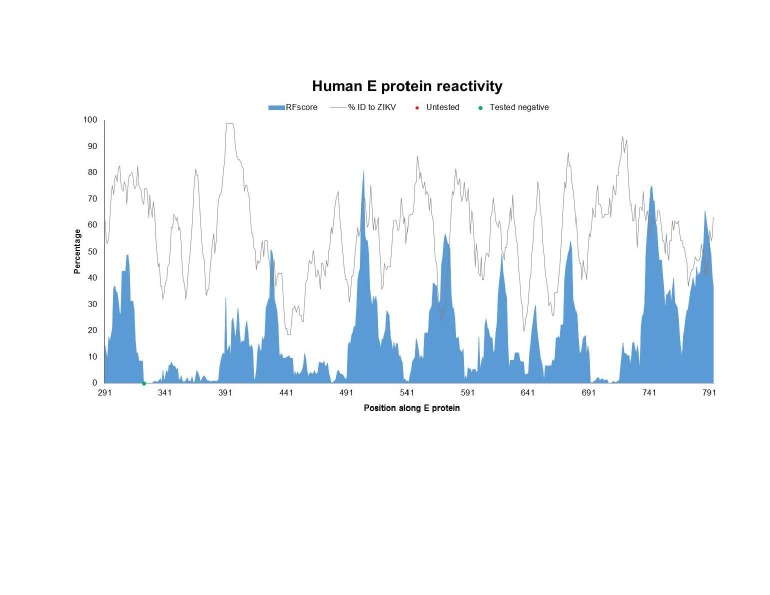
**Fig. 1B: **The Flavivirus sequence identity and human antibody response frequency visualized along the E protein. The sequence identity data (grey) shown represent a running average (window of 9 aa) of sequences for reference Flaviviruses to ZIKV, while the response frequency scores (blue) represent individual residues. Red and green dots along the x-axis represent untested and negative regions, respectively. Residues intervals along the x-axis of the polyprotein plot represent start sites of individual protein products in the polyprotein depicted also at the bottom of panel A.

**Figure 1c:**
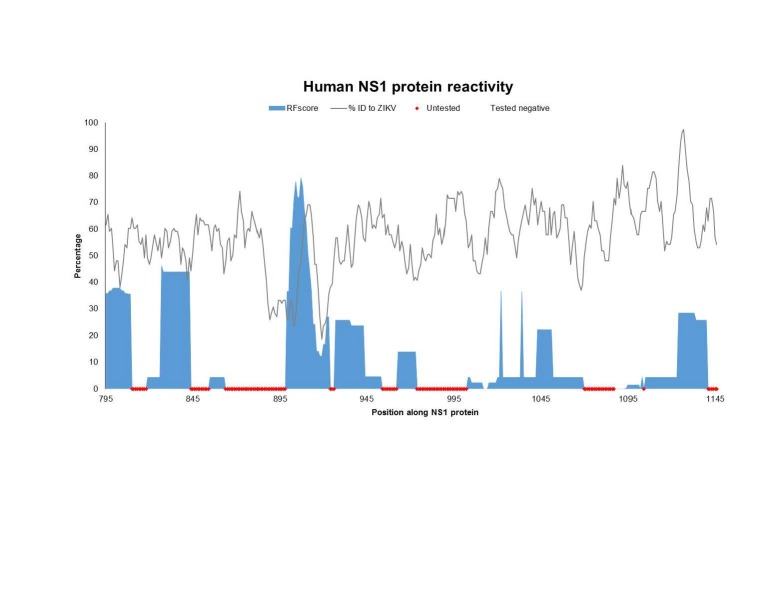
**Fig. 1C: **The Flavivirus sequence identity and human antibody response frequency visualized along the NS1 protein. The sequence identity data (grey) shown represent a running average (window of 9 aa) of sequences for reference Flaviviruses to ZIKV, while the response frequency scores (blue) represent individual residues. Red and green dots along the x-axis represent untested and negative regions, respectively. Residues intervals along the x-axis of the polyprotein plot represent start sites of individual protein products in the polyprotein depicted also at the bottom of panel A.

The epitope data include both linear and discontinuous determinants (monoclonal and polyclonal), and represent epitopes from all four DENV serotypes, WNV, JEV YFV, as well as TBEV. As expected, the majority of reactivity was observed for E and NS1, and close ups of those data are depicted in panel B and C, respectively. However, high responses were also observed for C and prM/M. Scant responses were noted for short regions within the other non-structural proteins, including NS2B, NS3, and NS5, with large sections of these proteins not being tested at all (red dots on x-axis).

Next, we wanted to specifically identify which of the major antibody response sites (those with higher than the average RFscore of 5.6% coincided with higher than average sequence identity to ZIKV (≥58% identity), thus representing conserved *Flavivirus* regions. We theorized that these sites would represent the greatest potential for antigenic overlap on ZIKV. **Table 3** provides a list of 27 regions along the ZIKV polyprotein that meet these criteria with six regions above 80% identify bolded. Not surprisingly, the greatest number of regions was identified in E (16 out of 27) and NS1 (8 out of 27). Interestingly, while several high antibody response sites were observed in C, overall sequence identity with ZIKV was quite low (45%), so that only two of the regions in C met the criteria for inclusion into the Table.

**Figure:**
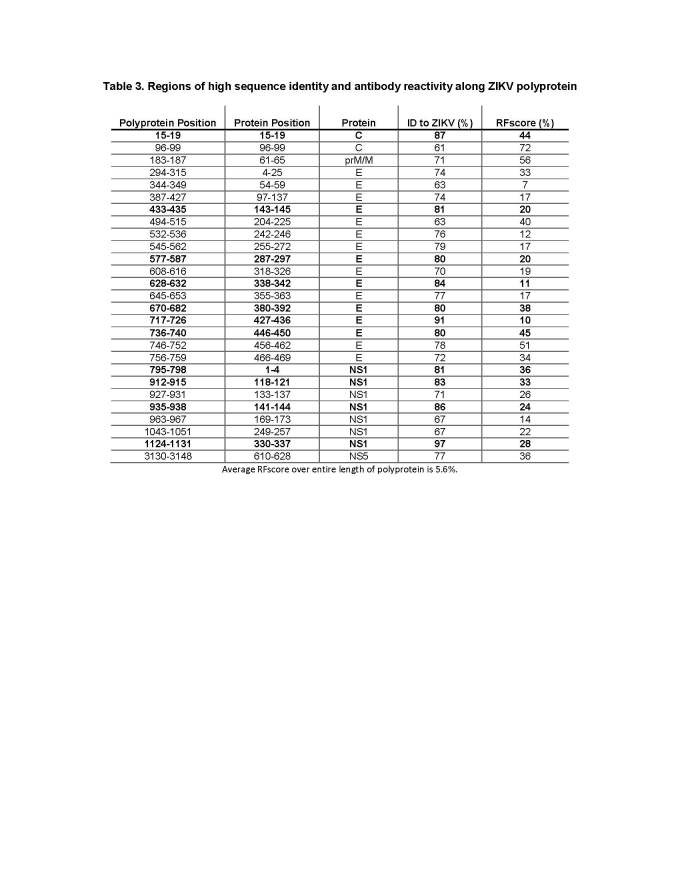
**Table 3.** Regions of high sequence identity and antibody reactivity along ZIKV polyprotein

Regions in ZIKV that have low sequence identity to other* Flaviviruses* are also of interest as they can potentially be a source of diagnostic epitopes allowing for the discrimination of responses induced by ZIKV from other *Flavivirus* infections. We thus selected regions which were targets of antibodies (RFscore >5.6%), but with low (<35%) sequence identify to ZIKV (**Table 4**). A total of 18 regions were identified that showed a more diverse distribution, with one region from NS3, two each from C and NS2B, three from prM/M, four from NS1, and six from the E protein.

**Figure:**
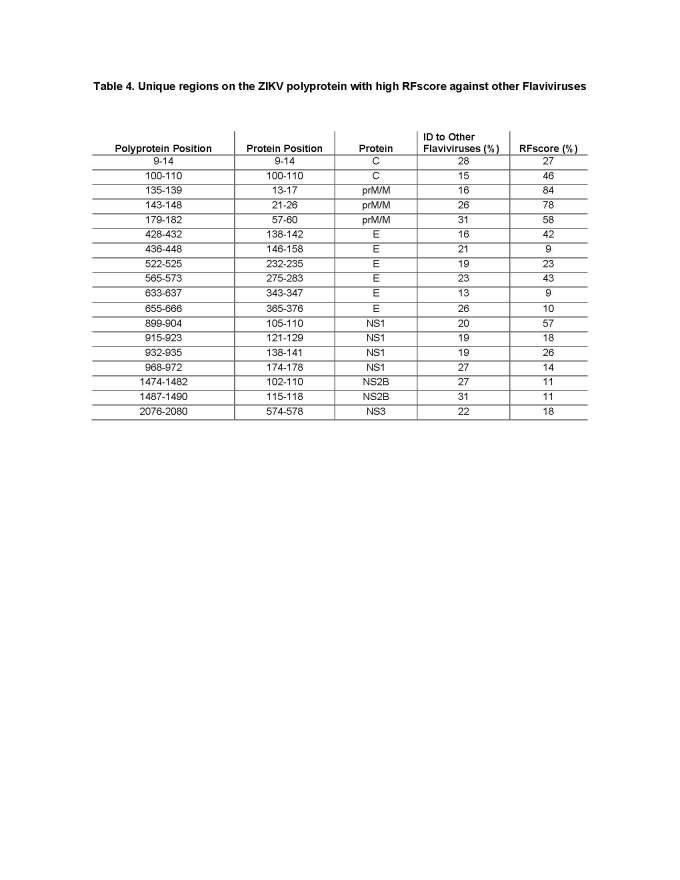
**Table 4.** Unique regions on the ZIKV polyprotein with high RFscore against other *Flaviviruses*

Structural analysis of ZIKV E and NS1 proteins. To analyze the epitope data with respect to the 3D structure of their source antigen, we used the recently published crystal structures for E (PDB 5IZ7)^12^ and NS1 (PDB 5IY3)^13^ to display the RFscores (**Fig. 2A, Fig. 2B**). Analysis of these data revealed sites, such as the fusion loop epitope (98-110), that are highly conserved, surface exposed on the partially mature virion or as a result of breathing^31^^,^^32^ and frequent antibody targets (high RF score). The 3D rendering for NS1 revealed greater distinction among the protein’s different regions for response frequency. Regions of high to intermediate response frequency were present on the wing, β-ladder and β-roll domains.

**Figure 2a:**
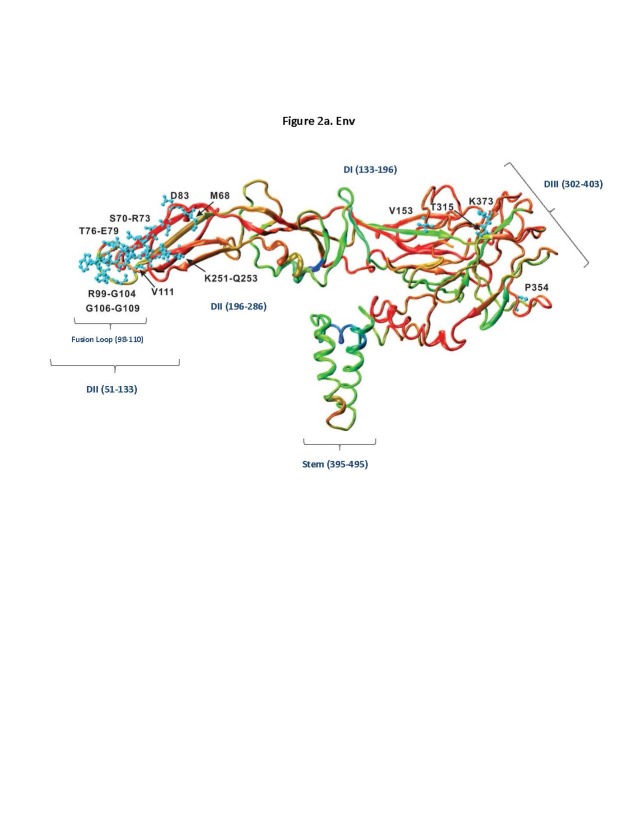
**Fig. 2A:** 3D rendering of RFscore mapped on the ZIKV E protein using PDB structure 5IRE showing functional Flavivirus epitopes (neutralizing and/or protective) defined in humans and mapped onto the corresponding locations on ZIKV E protein. Colors represent high to low RF scores, where (blue>cyan>green>yellow>orange>red), where blue is highest and red is lowest.

**Figure 2b:**
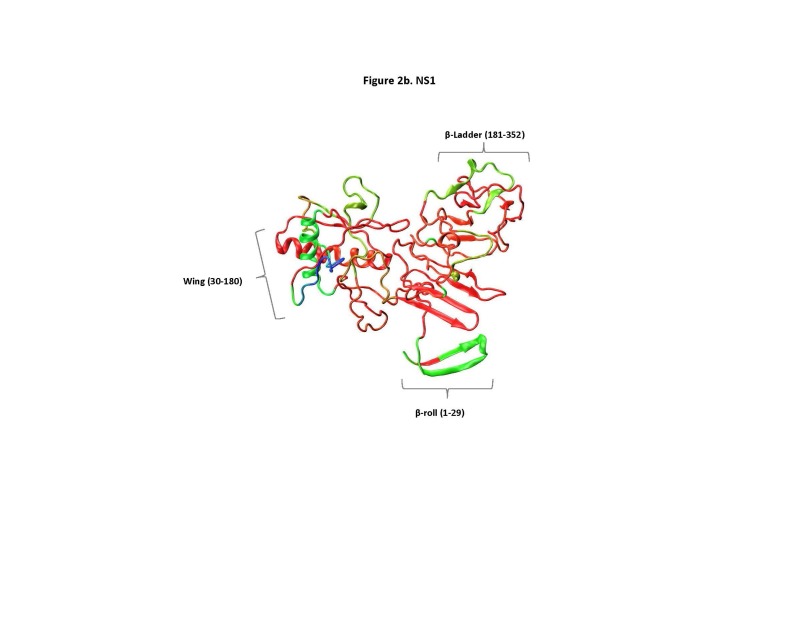
**Fig. 2B:** 3D rendering of RFscore mapped on the ZIKV NS1 protein using PDB structure 5IY3. Colors represent high to low RF scores, where (blue>cyan>green>yellow>orange>red), where blue is highest and red is lowest.

Next, we wanted to examine if either solvent-accessibility of residues or their conservation among Flaviruses was significantly correlated with the observed RFscores. Solvent-accessible surface area scores (SASA) were calculated for the ZIKV E and NS1 proteins based on the available crystal structures, to identify regions that are surface exposed versus those that are buried within the structure. We then evaluated the data for both antigens to determine if there were any statistically significant correlation between RFscore and SASA, sequence ID and SASA, as well as RFscore and sequence ID. We found no significant correlation among these variables. Given the importance of surface accessibility for antibody recognition, we hypothesize that the expected correlation was not found because of structural complexities (numerous in vivo conformational states of the antigen) not take into account in our analysis.

Interactive 3D renderings of E and NS1 showing all three data sets (RFscore, SASA and Sequence ID values mapped per residue) are available for viewing using the following links: [http://moles.liai.org/zikaEnv.html and http://moles.liai.org/zikaNS1.html].

**Analysis of functional antibody epitopes in ZIKV**

The analyses above focused on antibody epitopes recognized in human hosts and their likely cross-reactivity among *Flaviviruses*, which is of particular relevance for the design of sero-diagnostic tools. For vaccine design on the other hand, is desirable to know if antibodies are functional, meaning that they can inhibit or prevent an infection. We thus selected a subset of epitopes from the dataset above (Supplemental Table 2) which were defined in functional assays, namely in vitro neutralization, antibody-dependent cellular cytotoxicity, complement-dependent cytotoxicity and all in vivo challenge assays (protection from, survival from challenge, challenge decreased disease and pathogen burden after challenge). A total of 77 such functional epitopes was identified from the entirety of human antibody epitope data. A significant fraction of the epitopes (59 of 77) was contained within the E protein. Those with sequence identity with ZIKV and were mapped onto the ZIKV E PDB 5IZ7 structure (**Fig. 2A**). The vast majority of human epitopes mapped within DII and DIII of E.

We next sought to broaden the scope of this analysis to include non-human hosts, as a significant portion of functional *Flavivirus*-specific antibodies have been defined in animal models. Thus, we performed a separate analysis of the *Flavivirus* antibody data contained in the IEDB to include epitopes derived from all hosts, but as above, included only those assays associated with defining functional antibody activity. This broader analysis identified 290 unique epitopes (positive) derived from humans, horses, chimpanzees, macaques, pigs, rabbits and mice. Supplemental Table 3 lists these epitopes with detailed annotation for reference information, epitope sequence, source and position, antibody name (if provided) and isotype, assay type and cross-reactivity (if known), along with the epitope’s percent identity to ZIKV (Rio-U1 reference). Not surprisingly, a significant fraction of the epitopes was contained in the E protein. Of particular interest in this list of functional epitopes are those that are found conserved in ZIKV with high sequence identity, as it is more likely that a conserved site will have the same functional property. Indeed, 84 epitopes with ≥80% sequence identify were identified, making these targets of specific interest for vaccine development. The subset of functional antibody epitopes with ≥80% sequence identity to ZIKV is also provided in Supplemental Table 3**.**

***Flavivirus* T cell epitopes in the IEDB**

In addition to antibody epitopes, the IEDB also contains a significant number of T cell epitopes identified in *Flaviviruses*. To date there are 2,857 T cell epitopes from *Flaviviruses* contained in the IEDB, the majority of which were defined in humans with (2,181 epitopes), which are provided in Supplemental Table 4. The majority of human T cell epitopes were identified in DENV (1,613 epitopes), followed by YFV (187), WNV (165) TBEV (135) and JEV (83).

Using the same pipeline described above for the antibody epitope analysis, we sought to map all *Flavivirus* T cell epitope data reported to date onto the reference ZIKV antigen. For this analysis, we considered CD8+/class I and CD4+/class II responses separately, as processing and presentation of these epitopes is a result of separate pathways. Furthermore, we separated out two types of exposure to the *Flavivirus* antigens, namely vaccine studies in which participants received administered antigen in the form of commercial or experimental vaccine (**Fig. 3A, Fig. 3B**), and natural exposures, which imply presence of a live, replicating virus such as in the context of clinical disease, or of sero-positive individuals from travel/living in endemic regions (**Fig. 4A, Fig. 4B**).****

**Figure 3a:**
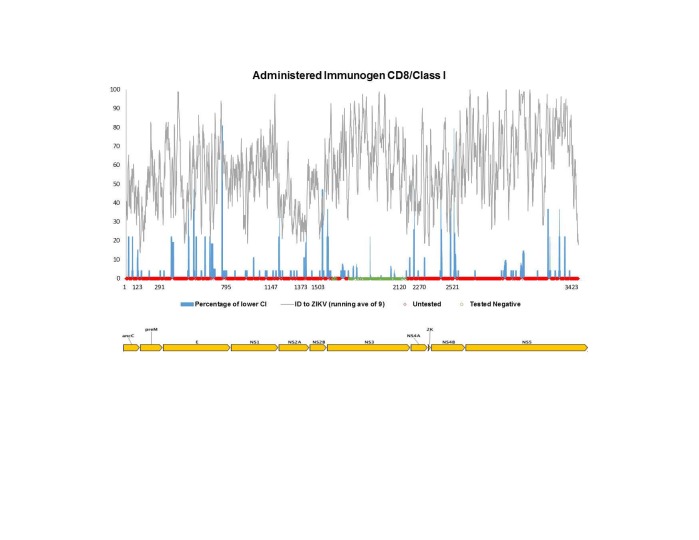
**Fig. 3A:** Flavivirus sequence identity and human T cell reactivity visualized onto the ZIKV reference genomic polyprotein for CD8+/class I responses to administered antigen (experimental or commercial vaccines). The sequence identity data shown (grey) represent a running average (window of 9 aa) while the RFscores (blue) represent individual residues. Red and green dots along x-axis represent untested and negative regions, respectively. Residues intervals along the x-axis represent start sites of individual proteins.

**Figure 3b:**
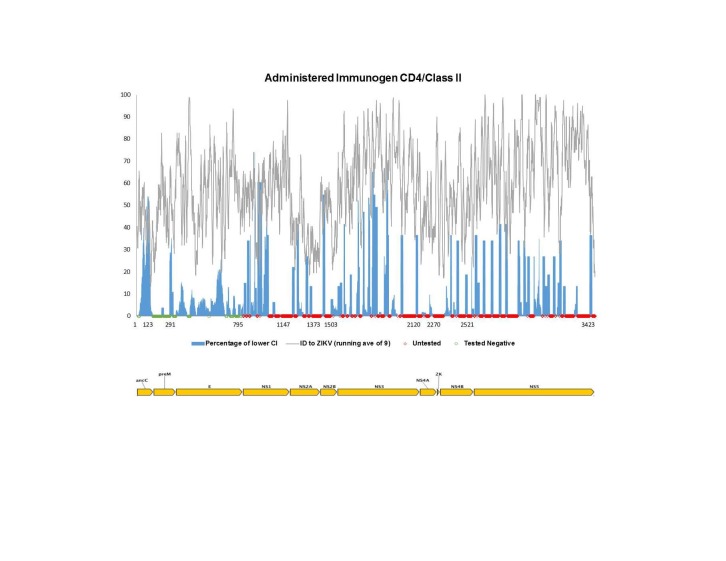
**Fig. 3B:** Flavivirus sequence identity and human T cell reactivity visualized onto the ZIKV reference genomic polyprotein for CD4+/class II responses to administered antigen (experimental or commercial vaccines). The sequence identity data shown (grey) represent a running average (window of 9 aa) while the RFscores (blue) represent individual residues. Red and green dots along x-axis represent untested and negative regions, respectively. Residues intervals along the x-axis represent start sites of individual proteins.

**Fig. 3A**and**Fig. 3B** show human T cell responses following vaccination. Such records are identified in the IEDB as having an ‘Administered Immunogen’ in contrast to a ‘Natural Exposure’ to an immunogen. Here, the immunizing agents defining class I MHC reactivity included the Yellow Fever Virus 17D vaccine, ChimeriVax-WN02, a chimeric West Nile/Yellow Fever (WN/VF) live-attenuated virus and experimental live-attenuated DEN (serotypes 1-4). Vaccines defining class II MHC responses included the Yellow Fever Virus 17D vaccine, experimental live-attenuated DEN (serotypes 3 and 4), the inactivated JEV vaccine (IXIAR and JE Vax) and the inactivated TBEV vaccine (FSME-Immun and FSMA-Immun). **Fig. 4A**and**Fig. 4B** show reactivity following natural infection, and included subjects with DF, DHF, West Nile fever, Japanese encephalitis, TBEV and WNV encephalitis, as well as healthy, sero-positive subjects. These data also represent numerous geographic locations, including S. North and South American, Asia, Southeast Asia and Europe.

**Figure 4a:**
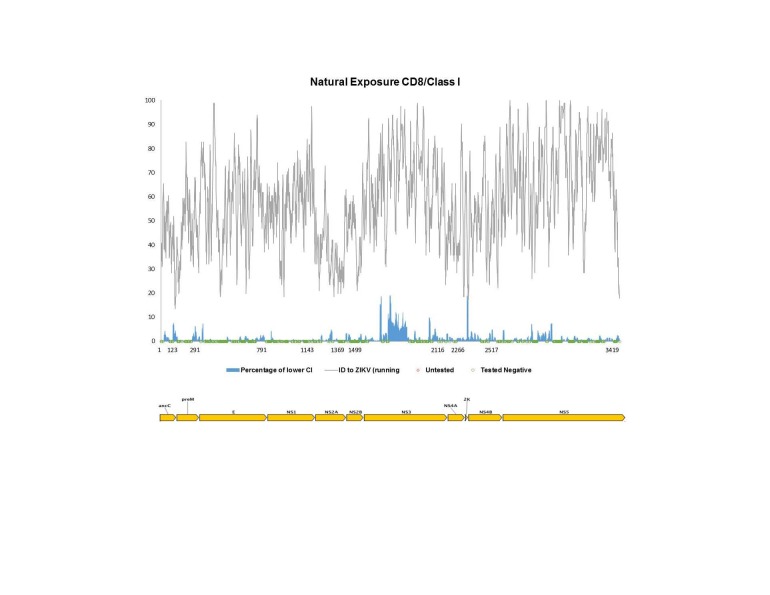
**Fig. 4A:** Flavivirus sequence identity and human T cell reactivity visualized onto the ZIKV reference genomic polyprotein for CD8+/class I responses to natural exposure (documented DF, DHF, DSS or living in/travel to endemic region). The sequence identity data shown (grey) represent a running average (window of 9 aa) while the response frequency scores (blue) represent individual residues. Red and green dots along x-axis represent untested and negative regions, respectively. Residues intervals along the x-axis represent start sites of individual proteins.

**Figure 4b:**
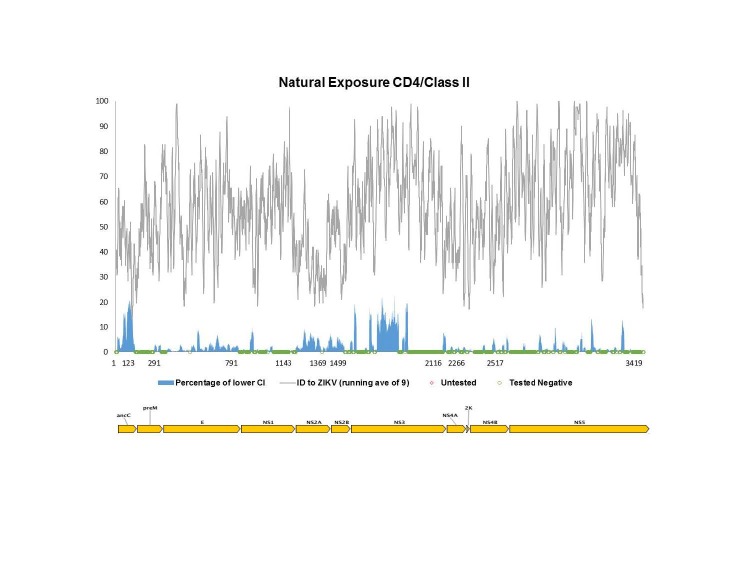
**Fig. 4B:** Flavivirus sequence identity and human T cell reactivity visualized onto the ZIKV reference genomic polyprotein for CD4+/class II responses to natural exposure (documented DF, DHF, DSS or living in/travel to endemic region). The sequence identity data shown (grey) represent a running average (window of 9 aa) while the response frequency scores (blue) represent individual residues. Red and green dots along x-axis represent untested and negative regions, respectively. Residues intervals along the x-axis represent start sites of individual proteins.

For both vaccination and natural infection we observed broader coverage in terms of the total number of reactive residues for CD4/class II compared with CD8/class I, as well as higher overall response frequencies for class II MHC antigens. However, when analyzing gaps along the x-axis, that is, determining whether these regions represent untested (red) versus negative or unreactive (green) residues, we found that whereas the majority of gaps present in the context of vaccination represent untested regions, the reverse was true in the context of natural infection; in the latter case, all gaps were shown to be unreactive (tested negative). For vaccination, only two significant unreactive regions are shown with NS3 for class I and within prM for class II MHC epitopes, while large portions of the polyprotein marked in red denote untested regions. This implies that the map for immune responses post-vaccinations in Figure 4 is potentially incomplete.

**Table 5**provides a list of regions demonstrating higher than average RFscores and high sequence identity (≥58%) for CD8+/class I and CD4+/class II MHC responses. Highlighted in this list in bold font are regions with ≥80% sequence identity with ZIKV proteins, most of which reside within NS3 and NS5, irrespective of immunization category (vaccination or natural exposure). This supports the idea that immune responses in non-structural proteins of *Flaviviruses* have the potential to be broadly cross-reactive. This should be taken into considerations in the design and evaluation of candidate vaccines for their capacity to induce T cell responses to *Flaviviruses* in general and ZIKV in particular.

**Figure:**
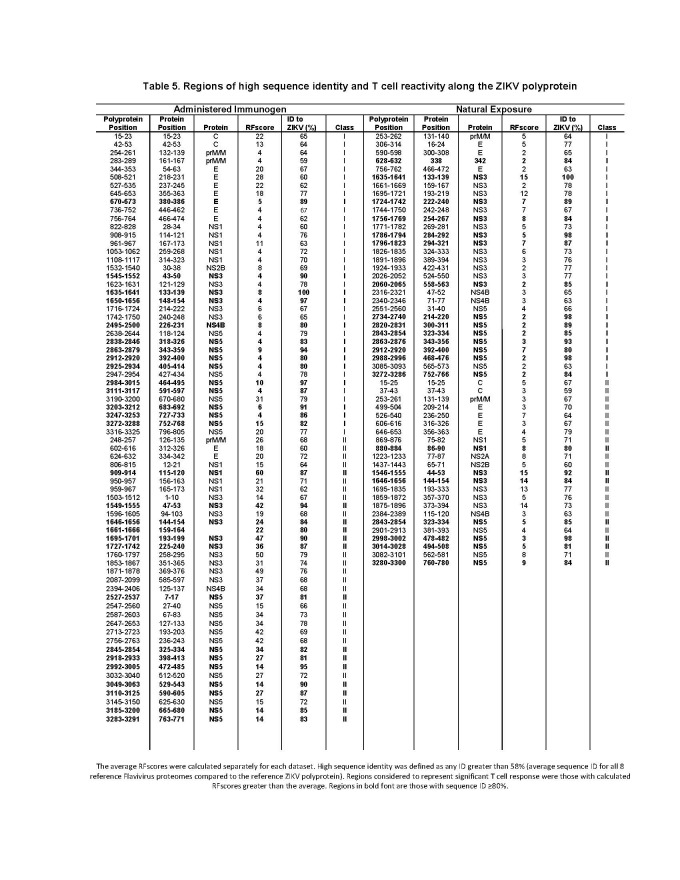
**Table 5.** Regions of high sequence identity and T cell reactivity along the ZIKV polyprotein

## Discussion

****The current outbreak of ZIKV infections has resulted in a massive effort to accelerate the development of ZIKV specific diagnostics and vaccines. These efforts would benefit greatly from the definition of the specific epitope targets of immune responses in ZIKV, but given the relatively recent emergence of ZIKV as a pandemic threat, few such data are available. We used a large body of epitope data for other *Flaviviruses* that was available from the IEDB for a comparative analysis against the ZIKV proteome in order to project targets of immune responses in ZIKV and their potential properties.

The most common approach to diagnose if a person has been infected with ZIKV in the past has been to test by serology if there are antibodies present in the patient’s blood that recognize ZIKV antigens. Several such serological tests exist that probe for the presence of anti-ZIKV antibodies. However, such tests are often unable to distinguish between ZIKV infections and infections with other *Flaviviruses*, due to the large potential for cross-reactivity^27^^,^^28^^,^^29^^,^^30^. Our epitope based analysis provides guidance on which components of ZIKV are most likely unique targets of ZIKV specific antibodies (which should be included in a ZIKV specific diagnostic test), and which targets in ZIKV are most likely to be cross-reactive with other *Flavivirus* species (which should be excluded in a ZIKV specific diagnostic test).

In addition to antibody-based tests for ZIKV infection, it is also conceivable to design T cell response based diagnostics. T cell based tests are a now routinely applied in the clinic to diagnose infections, for example with M. tuberculosis using the Quantiferon-Gold-test^33^. We found that T cell responses in *Flaviviruses* following natural infection targeted primarily non-structural proteins and that the same regions were found to be conserved in ZIKV. Thus, to develop T cell based diagnostic assays specific for ZIKV infections, it will be necessary to experimentally determine the cross-reactivity of T cell epitopes in other *Flaviviruses*.

In terms of vaccine and immunotherapeutic development, the challenge for ZIKV is to induce a functional immune response characterized by potently neutralizing antibodies^34^^,^^35^. From our analysis of antibody data, we found that there are a number of functional epitopes (neutralizing and/or protective in vivo) on the E protein showing high sequence identify with ZIKV (80-100%), further supporting that inducing antibodies against the ZIKV specific epitopes on this major antibody target could induce protective immunity. Moreover, recent work characterizing ZIKV-immune sera from humans has shown that ZIKV exists effectively as a single serotype36^36^. This contrasts with DENV and suggests that it should be feasible to identify conserved protective epitopes across ZIKV strains from disparate regions. Similarly, recent works have shown that quaternary epitopes (those spanning more than on domain on Env) are important targets of serum neutralizing antibodies in DENV2 infected and vaccinated subjects^22^^,^^37^. However, this phenomenon is out of the scope of the current analysis, as the orientation of antigens in different *Flavivirus* species has not yet been sufficiently resolved. Further, it is important to emphasize that while this study and others have shown the potential for cross-reactivity among co-circulating *Flavivirus* species and ZIKV, to date there are no definitive publications documenting ZIKV-related ADE in humans. Nevertheless, this remains a critical question requiring further investigation given the breadth of the current outbreak and its overlap with numerous endemic *Flavivirus* regions.

From the analysis of existing T cell epitopes we found that there are few recognized T cell epitopes in the E protein, rather a significant portion of T cell reactivity targets non-structural proteins NS3 and NS5. Indeed, several studies defining the targets of human CD4+ and CD8+ T cell responses against DENV have suggested that the major targets of CD8+ T cells include the highly conserved non-structural proteins, NS3, NS4B, and NS5, whereas CD4+ T cells target both structural and non-structural antigens, mainly capsid, NS3 and NS5^38^^,^^39^^,^^40^^,^^41^^,^^42^^,^^43^^,^^44^. If a combined immune response of antibodies and T cells is necessary to prevent Zika infections, then it will not be sufficient to limit vaccine design to prominent antibody targets such as the E protein, but it will also be important to include conserved regions within non-structural antigens.

It is important to note that while our analysis identifies many different domains within ZIKV antigens as potential B cell or T cell epitopes based on sequence identity, it is more difficult to explicitly single out specific domains that we can say with certainty are critical to develop Zika virus specific diagnostic tools or definitively name those that will be immunologically potent for ZIKV vaccine development. Since these data were generated base on strong inference rather than experimentation we have interpreted the findings keeping in mind that the threshold of cross-reactivity varies, not only between different antigens, but also among different effector cell responses. That is, while the exact sequence identity necessary for biologically relevant for antibody cross-reactivity may not be the same for class I/CD8+ and class II/CD4+ T cell reactivity. Indeed, the threshold for cross-reactivity for antibody, CD4 and CD8 T cell responses is not fully characterized among all Flavivirus antigens in humans, and must ultimately be determined empirically. Our objective in this analysis was to provide the Flavivirus community a framework based on the cumulative epitope data for this critical ongoing work.

## Competing Interest Statement

The authors have declared that no competing interests exist.

## Corresponding Author

Kerrie Vaughan: kvaughan@lji.org

## Data Availability Statement

All relevant data are included in the manuscript.

## Appendix

Supplemental Materials

**Figure:** **Supplemental Fig. 1:** The degree of sequence conservation for consensus residue at each position for ZIKV polyprotein. The red line represents the sequence identity and the blue represents the percentage of residue deletions (if any). Deletions present at N- or C- termini are possibly the result of incomplete sequences and thus may not have biological significance.
